# Infants

**Published:** 1874-03

**Authors:** Alexander S. Von Mansfelde

**Affiliations:** Jefferson, Illinois


A MONTHLY RECORD OF

*Medicine, Surgery and the Collateral Sciences.*

EDITED BY J. ADAMS ALLEN, M.D., LL.D.; AND WALTER HAY, M.D

VOL. XXXI. — MARCH, 1874. — NO. 3.

### Original Communications.

ARTICLE I.—*Infants. Their Food and its Digestion.* By ALEXANDER S. VON MANSFELDE, M.D., Jefferson, Illinois.

"The hand that rocks the cradle, is the hand that rocks the world.

However true this beautiful verse may be, it is outlived by another, which, though less rythmical, is so much the more undisguised by poetical license. "*The hand that nurses the infant, is the hand that digs its grave.*" And if this hand is not directed by a mother's divine love for her offspring, this occupation reaches a climax, the comprehension of which leaves the observer horror-stricken. In the city of Munich, statistics show, that of one hundred deceased infants, eighty-eight did not have the mother as a nurse. Classifying the causes of infantile mortality, we find that a certain amount of *carelessness*, however harsh this may sound, takes the initiative, and out of this the tool is wrought that does the mischief, namely, *entire ignorance of the laws that govern the welfare and the growth of the infant man.* This lack of knowledge is by no means confined to the nurse of the infant, but even the physician of this enlightened age carries a little of it upon his shoulders, only too often the *offspring of superficiality*, not of learning, perhaps, but begotten by a routine practice.

In the latter part of July I was called to see Blanche, the three months old daughter of Mr. F., of Chicago.

The parents of the patient, quite intelligent people; father's occupation, bookkeeper; of nervo-sanguine temperament, not very strong, but of a bodily composition betokening a close knit frame—in fact, made to endure. The mother a perfect picture of health, well capable of giving birth to healthy children, but more so, qualified to nurse them.

Her confinement was natural and easy; the babe was well developed, and manifested tokens of vigorous life. The mother nursed the child herself the first few weeks after the confinement; but all at once, without failure of health, she lost the use of the breasts. The physician that assisted in her confinement, resorted to all known means to restore the action of the glands, but without avail; the child had to be fed, and as the milk of *one cow* could not be had, and the milk of the milkman was of doubtful composition, *condensed milk* was the *sine qua non* of the physician. This was duly bought by the dozen, and artificial nursing commenced. Everybody was satisfied but the baby. This, having been a good child until now, manifested its inborn badness by restlessness, crying, and all the signs of ill-temper and body. The physician was called, and prescribed for the baby. This manifesto of medical qualification was carried on to such an extent, that we found at the time of our visit, a good selection of all the mixtures that a good pharmaceutist can boast of, and above all, a baby that was taking pepsin and bismuth powders, *one every three hours*, the last prescription of the physician, and this would surely have been the last one, for the child was dying of STARVATION.

Three months old, the child weighed even less than at birth, about seven pounds; the skin hung loosely around the limbs, and had that peculiar cold and wet touch found in the wasting diseases of children. We considered it a case lost; but with a resolution, long since formed, centred in the sentence, "*where there is life, there is hope*," we told the parents we would try our best.

An error surely manifested itself here. Who committed it? What was it?

Not being able to find the cause of the non-secretion of milk, we directed our attention to the food and general nursing of the baby. *The child was not clad warmly enough for its condition.* Every practitioner knows, that in the wasting diseases, *i. e.*, in their decline, the natural heat fails very rapidly, or, better, its production is not proportionate to the wants of the organism; assistance should be procured by artificial heat. This we acted upon at once.

Next we observed (and who has not with us?) that *the child had by no means the solitude and rest that even healthy children always should have.* This is lacking in the first children of our better classes, and is a greater cause of the death of their offspring than most people are aware of. Are they the divine gifts the Creator has laid into the hands of parents for safe keeping? Does the mother try to be the guardian angel of her infant babe? In nine out of ten cases she has not even a thought of her grave charge. Used to play with dolls, until she marries, and tired of her mustached substitute for the same, kind nature loses its wisdom by presenting her with a babe, a living doll! How she enjoys to play with it! But this living doll has one great fault; it is not made of leather. It is soon worn out; the least disorder, and that soon will come, crazes the already surcharged nervous system, and the little one will soon be the victim of its mother's ignorance in this direction. We ordered a cradle for our patient, had it well prepared, and put it, with the baby, into an off-hand bedroom, the air being good in the neighborhood, and had it guarded from all noise of the house and its surroundings as far as possible.

*Next, we mistrusted the food the child received*—CONDENSED MILK! Now, with all regard for the pharmaceutical and culinary so-called fancy preparations, we touch them with a certain amount of misgiving. Think of the elixirs! What are they? A nice thing for a physician who does not know how to prescribe, but surely not a good equivalent of medicine for the patient. It is the same with artificial food, and above all, *condensed milk.* We confess our ignorance of its preparation, but that the article in question was not casein 68, fat 38, sugar 30, and salts 6 parts less more or less of its 860 parts of water, we are satisfied. We knew that some of the water had gone, but with it also some of the fat, and to the market, in the shape of butyric acid combined with the haloid, oxide of lipyl. *It was the absence of fat that caused the trouble in this case, and will cause it again and again.*

At last we eyed the prescription: pepsin and bismuth, a powder every three hours. A good prescription for indigestion, we admit; but the digestion of what? Was it to aid the food to come? The baby received the powders repeatedly upon an empty stomach. This surely was not the intention of the physician, but the mother followed his direction, a powder every three hours, and they did their work. The consequence of a superficiality, caused by routine practice. It was not ignorance, for this member of the profession is well versed in the *science* of medicine.

We saw this same child fed upon warm *cows' milk from the milkman*, lying in its comfortable cradle, warmly dressed and covered, away from the noise of the house and the street, freed from pepsins and bismuth, sleep quietly for several hours, the first time for some days, the mother affirmed, and with its sleep came the angel of health, and brought joy to the parents, life to the little sufferer, and to us a great lesson.

Afterward we resorted to a nurse (wet), and with some degree of care, the child was saved; it weighs now its ten pounds, and is in all respects well.

The natural food of the infant is milk from its mother's breast, the composition of which is, casein 35, fat 25, sugar 48, salts 2, and water 890 parts. (Simon). Upon this the babe rapidly grows, if it be furnished in good quality and quantity. But if the iron hand of fate or the cruel grasp of fashion deny this natural food, an artificial one must take its place, and the best of our craft have united on diluted cows' milk, with the addition of a little sugar, as the best substitute.

This dilution and admixture, to reduce the percentage of the nitrogenized elements, the casein, and render it more suitable for digestion and assimilation. Wells' Chem., p. 488. Now, we dare to assert that neither the first nor the last is true. Because,

1. By the addition of water no dilution of the casein takes place.

2. It is not physiological to give food, of whatever description, in a highly diluted form.

3. The digestion and assimilation of casein is not assisted by the addition of sugar.

4. It is the addition of oil that will make the artificial food a healthy substitute for the mother's milk.

As a revolution in this direction sooner or later must take place, the JOURNAL will kindly furnish us space for the maintenance of the above statements. We may be wanting in revolutionary qualification, but perhaps may be able to make some noise toward scaring things into better times for the infantile world.

There are found in the milk of woman about 35 parts of casein to a thousand; whereas, in that of the cow, no less than 68 parts are contained. (Simon). Now take 100 scruples of fresh cows' milk, about as much as a healthy child takes at a time, and mix this with 100 scruples of water, does this addition lessen the amount of casein in the fluid? It is simply a dilution of all the solid ingredients of the milk. In order to have the child get the benefit of the 100 scruples of milk, it has to be given in a bulk of 200 scruples, rather too much for one meal, and if it is given, it gets all the casein contained in the 100 scruples of milk, and if only half is given, it gets the casein of 50 scruples of milk, but existing in this milk in the ratio of 3½ parts to a hundred, in spite of 50 or 100 scruples of water superadded. *This water separate*, as it exists *out of the body*, *is separated at once in the stomach*, *leaving the casein intact.*

As to our second assertion, we are inclined to believe little is necessary to be said. For it is not only known to physiologists that an unnatural dilution of food retards its digestion in the stomach, and as this organ in the human family is by no means created to contain fluids in quantities, these rapidly egress into the intestinal canal, where they are acted upon by the admixture of bile, pancreatic fluid and intestinal juice, to be further prepared for absorption and assimilation; but also the pathologist and practitioner know the dangers which the partaker of food incurs, when protein compounds are not acted upon sufficiently by the gastric juice to insure their absorption by the intestinal gland apparatus. One more reason, and we are satisfied a very tenable one, is the fact, sustained by physical laws, that the larger the dilution of certain bodies, the heavier the resulting precipitate will be, (think of the heavy and light magnesia carbonates); and that the texture of the coagulum is the index to the facility with which this is changed by the gastric juice into peptones. Now, suppose that we are sustained by the above mentioned scientists, what good does the additional water do, which quickly passes into the intestinal canal? It induces a closer union of the casein molecules, which is already the case in cows' milk, making its digestion more difficult.

In order to do justice to our third assertion, we have to consider the digestion of casein and of sugar.

CASEIN, identical with the *legumin* of the seeds of certain plants, and the *crystallin* of the lens of the eye, having a chemical composition of $C_{40} H_{31} N_{10} O_{12}$, (Mulder), but most likely a conglomerate of different protein bodies, is the nitrogenous principle found in the milk of the mammalia. It undoubtedly takes its origin in the blood, from which it is separated by the glands of the breast; but the exact derivation must be a mystery, until albumen and casein receive their proper places through researches in physiological chemistry. As to the use of casein, no doubt can be entertained. It is the desideratum for the growth of the young animal, which not only derives the pabulum for its soft structures from it, but also the material for its bony frame, through lime salts, which are abundantly present in the casein precipitate.

Milk enters the mouth of the infant at a temperature of bodily warmth (98° Fahr). Here it is mixed with the secretions of the buccal cavity, called saliva. Has this any influence upon the digestion of casein? We say, most positively, no! It may exert a relaxing influence upon albuminoid food, like that of warm water; this is all, and surely not needed, when the highly diluted casein is eaten.

After having entered the stomach, the milk loses its identity, the casein is precipitated from the whey, which latter takes with it the sugar, some of the salts, and traces of oil. By the peristaltic motion of the stomach the coagulum is reduced to a pulpy mass, being at the same time mixed with the gastric juice; *but this division does not take place until the greater part of the whey has found its way into the duodenum.* The rapidity of division is of necessity also regulated by the texture of the precipitate, as above stated. After the solution of the coagulum, it does not present the same chemical character as that of the casein solution, milk. It is not precipitated by acids and metallic oxides, as the latter; it is no more *casein* in solution, but *casein peptone* in solution. The latter owes its formation either to a catalytic influence of the gastric juice, *i. e.*, its pepsins; that is, it has changed itself, not losing its chemical equivalents, but a different grouping of its atoms has taken place, or it has formed a pepto-casein-salt with the earths that are largely present in casein; the former having been acted upon by the free acid always present shortly after digestion commences in the stomach. Thus prepared, the casein compound slowly enters the intestinal canal, and in this locality its absorption is further facilitated by the action of the intestinal juices upon it, whose power upon it closely resembles that of the gastric secretion.

SUGAR, $C_{12} H_{11} O_{11}$, as it is found in sugar cane; is also contained in fruits, chylariose $C_{12} H_{12} O_{12}$; in milk, as lactine $C_{12} H_{12} O_{12}$; in different species of agaric; in the mountain-ash, as sorbit $C_{12} H_{12} O_{12}$; in the substance of muscle, as pnosit $C_{12} H_{16} O_{16}$; in manna, as mannite $C_{12} H_{14} O_{12}$; in the acorn, as quercit $C_{12} H_{12} O_{10}$; in the protococcus vulgaris, as phycit $C_{14} H_{16} O_{12}$; in the eucalyptus manniferæ, this sugar has the same composition as cane sugar, but with a double grouping of its elements, $C_{24} H_{22} O_{22}$; in the liquorice root, as glycyrrhizin $C_{16} H_{12} O_{6}$. All these, and several less noticable products of the vegetable kingdom, are classed under the name of glucosides, glucose, or grape sugar $C_{12} H_{14} O_{14}$, being the standard product of the change which they undergo when introduced into the animal organism.

Sugar is not acted upon by the buccal secretions, excepting perhaps some of the more complicated glucosides, but even these can only be changed by long standing, with the saliva at a high temperature; could not, therefore, be acted upon by this medium in the short space of time allotted to their presence in the mouth, since the action of the saliva is positively interfered with by healthy gastric juice. Sugar lies actually inert in the stomach, excepting its solution and transference to the duodenal canal, from whence the fluids regurgitate, as observers have shown, to favor this transmission.

It is the intestinal tract that favors the digestion and absorption of sugar; soon after taking it, it finds its way into the intestinal canal, which it quickly traverses as far as the first half of the large intestine, from whence it is absorbed *in naturam*, or is changed in the small intestine to lactic and in the large intestine to butyric acid; either of which, combining with the alkalies, the salts of which are found in the secretions of this locality, are also absorbed and carried into the economy to answer their purpose.

But observe, this acidity and chemical change leave a reddened condition of the intestinal membrane, an inflammatory indication, not even noticed, when chloride of sodium is substituted for sugar.

Are we justified to assert, that the addition of sugar to the diluted milk is not only of no help to the digestion and assimilation of casein, or any other protein compound, but is even of harmful tendency, when taken in any quantity above the requirements of the organism?

Who will tell the limits of its healthy or abnormal action? Who, of any practice, has not observed the ill consequences or uncomfortable condition of infants who have been fed with overdoses of sugar, or, what is worse, with candies of unhealthy composition?

Before entering upon the final subject of these lines, we take leave to diverge therefrom, being justified by the preponderance of starchy elements that make up the so-called artificial foods for infants.

WE DECLARE THAT IT IS ILL PRACTICE *to advise or silently permit the use of starch, arrowroot, tapioca, sago, wheat flour, crackers, or any combination of these, into the numberless nuisances called Liebig's food for infants, royal patent food, etc.*

It is a scientific fact that starch, in order to be of benefit to the body of the infant, must be converted into glucose. Now, Dr. Sonsino has proven that starchy food is not digested in infancy, and that there really exists a physiological despepsia for starchy aliments, caused by the inactivity of the humors that concur in the digestive process.

Now, as starch is changed into glucose by the action of the saliva, being interrupted in the stomach, and again powerfully taken up by the pancreatic fluid, which mixes with it in the intestinal canal, and completed by the intestinal juices; and as the saliva, as well as the pancreatic fluid, have little or no power over this material in the first months of infantile life, the changing influence of the intestinal secretions being infinitesimally small; and as *post mortem* examinations have repeatedly shown that this is actually the case, pathologists having found the whole intestinal canal literally pasted up by starchy food; we cannot but wonder at the audacity with which men of learning dare to endanger the welfare and the life of the little ones intrusted to their care, by being silent upon this subject. If ignorance of mothers is a great cause of infantile mortality, why do not those who know bettei guide them in the right direction? *Knowledge is only of benefit when it is applied.*

Our fourth assertion, the novelty of which will be admitted, compels us for this reason, and for the sake of the importance of the subject, to be carefully minute. We hope that we will have the indulgence of the reader if we seem to repeat and state facts known to him already.

FAT is the link nature proposed between the inorganic and organic elements that constitute the animal body; it is present wherever life manifests itself, either in the growth of already existing beings, or in the propagation of the same. The radical of this hydrocarbon is called *lipyl* $C_3 H_2$, and the "*haloid base*," according to Berzelius, *oxide of lipyl* $C_3 H_2 O$. This base, combining with the fatty acids, form neutral salts, the fats, which by the addition of oxygen, assume an acid character, and separate into free acids and *glycerine*, the combination of which is $2 (C_3 H_2 O) + 4 H O$. That is, two equivalents of oxide of lipyl combine with four of water. The acids that most concern us are, margaric acid, $C_{34} H_{33} O_3 HO$; stearic acid, $C_{36} H_{35} O_3 HO$; oleic acid, $C_{36} H_{33} O_3 HO$; and butyric acid, $C_8 H_7 O_3 HO$, (or $C_8 H_8 O_4$).

The combination of these acids with oxide of lipyl form the most of the fats, and the ratio in which these salts combine is the measure of their consistency. The stearate being the most solid, the oleate being fluid, as also is the butyrin.

Mechanically considered, fat is a preserver of animal heat, and a protector of the delicate organs of the body; we only refer to the structures of the orbital cavity, which are, even in the emaciated, yet surrounded by a layer of fat; it also gives shape to the body. Lehmann expresses this very nicely; he says: "Fat is the cosmetique employed by nature to stamp the human countenance with the incomparable impress which exalts it far above all the other lower animals."

Fat is not acted upon by the saliva, nor has the gastric juice any influence upon it, except perhaps its separation from protein bodies. The intestinal canal is nature's workshop, where fat is prepared for absorption. The bile, the first of the intestinal juices it comes in contact with, seems not to exert a great influence upon its digestion; in fact, as it is more an excrement than a secretion, one would be warranted to assert that it had no other than a mechanical influence in the digestion of fat. The pancreatic fluid seems to have the power of emulsifying the fat, and thus by minute division hastening its absorption. This process takes place from the lacteals, favored by the moisture of its delicate membranes by the bile, as it would hardly be admissible that animal tissues, kept moist by aqueous humors, would admit of such filtration. Absorption is also assisted by the small organic muscles of the interior of the villi, by a pressure at intervals exerted by their contraction. But it is also proven that the capillaries of the villi take up small quantities of fat, and carry it directly to the circulation of the portal system, which is always richer in fat after a meal of fatty food.

Chemico-physiologically considered, fat is the fuel, according to Liebig's theory, the burning of which assists largely in the maintenance of bodily temperature. The heat of the body is, under different atmospheric degrees of warmths, almost stationary, excepting perhaps the young animal, in which the normal temperature is higher than in the adult, and which (what is very remarkable) is not capable of maintaining the equilibrium of warmth, as is the adult. A small child rapidly loses its normal degree of heat when under the influence of cold.

One reason more why young children should be carefully guarded against changes of temperature.

Examining the physiological role that fat plays, we quote Lehmann, Physiological Chemistry, vol. 1, page 236:

"I was long since led from theoretical grounds to regard the fat as one of the most active agents in the metamorphosis of animal matter, and this subjective conviction has since been converted into objective proof by numerous experiments and observations. After having found by experiments, regarding the fermentation of milk, that the process cannot be excited by albuminous bodies in saccharine or amylaceous fluids, *excepting with the co-operation of fat*, I next ascertained *that a certain, although small, quantity of*

*fat was indispensable to the metamorphosis and solution of nitrogenous articles of food during the process of gastric digestion.*"

"When we consider all the facts," continues Lehmann, on page 239, "we shall be almost involuntarily led to the conclusion that fat takes a highly important share in the most important, and at the same time the most mysterious, processes in the formation of cells and tissues. We cannot believe that fat is a mere incidental agent in all these processes; but we must rather regard it as of essential aid in the process of converting nitrogenous nutrient substances into cells and masses of fibres, in like manner as it co-operates in the processes of lactic fermentation and digestion; and it is probable that whenever a chemical equation, presenting the formation and function of certain cells, can be established, fat will constitute one of the integral factors. Indeed, it is impossible to believe, that in the vital activity of cellular action, fat should be without influence on the metamorphosis of the substances which it accompanies, and that, without reference to them, it should obey only its own affinities toward oxygen or an alkali."

In pathology we meet with fatty degeneration of the muscles, the glands, the liver and the kidneys, in either case followed by the most disastrous consequences. Here we also cite the fatty infiltration, taking place when fat is absorbed in quantities above the wants of the body, or the natural ratio is not used for the formation of bile, etc., caused by disease of the liver and other organs.

The lipomata, which favor the places where infiltration takes place, (excrescentiæ membranæ adiposæ of Margagni), are the consequences of like causes. In all carcinomatous growths, fat plays a great role, for the quicker the generation of the cancer cells, the larger the amount of fat present.

Or is perhaps the generation of these cells the consequence of an increased amount of fat, favoring the rapid development of compound nucleoli, or nuclei, into cells? How is it that cancerous growths always take their origin at the site of union of two distinctly different cell forms?

If the fat could be abstracted, would the growth of the cancer be interrupted, or altogether checked?

And just as the presence of fat favors new formation, so does its absence retard the same; we refer here to tuberculous deposits.

It is known, that in this pathological condition, it being retrogressive, the blood is poorer in fat than in any other diseased state.

Thus having traced the influences brought to bear on fat, and also its changes, and the alterations produced by the same under various circumstances, we are prepared to prove our assertion, *that it is fat which favors the digestion of albuminoid food, especially fibrine, in the food of the young.* It is a demonstrated fact that veal and lean pork are most difficult of digestion. Every housewife is aware of it, and who, in this country, has not had the experience that when these meats are prepared with a certain amount of fat, they, though yet heavy food, are well borne by the stomach, which otherwise would manifest its protest by discomfort, better felt than described?

Elsaesser has demonstrated that the milk of women is more easily digested because its fibrin-coagulate is of a more spongy character than that of cows' milk, which forms not only a denser coagulum, but the whole is of a more clotted texture.

If this physical condition is the cause, what then in the two milks is it that makes this difference? and is there anything in science that will alleviate the obstacle which prevents cows' milk being a good substitute for mother's milk?

Is it a lessened per centage of water in cows' milk that gives rise to the difference? The difference in thousand parts is from 10 to 20 only; why then advocate the addition of 990 parts of water? We cannot see the scientific deduction that lies in this admixture.

Is it the addition of sugar to cows' milk, that makes this similar to the natural food of infants? We have seen that this carbo-hydrogen compound has no influence at all upon the digestion of the protein bodies, nor is it acted upon by the secretions of the alimentary canal forcibly enough to warrant the conjecture, that by decomposition it may exert some influence upon stomach digestion.

*It is the difference of fat contained in the respective milks.* The milk of the cow holds 10.5 parts less fat than that of the human female, when compared with the casein found in them.

We instituted the following experiments:

1. We took 7½ drachms of fresh cows' milk, and added a half drachm of white vinegar, shaking the fluid before and after the mixture.

2. We mixed 7½ drachms of the same milk with twenty drops of sweet almond oil; then we added a half drachm of the same vinegar, shaking the compound well.

3. We added to three and three-fourth drachms of the same milk as much water, mixing it well with the former; then we added a half drachm of the same vinegar again, shaking it well.

The fluids were contained in two oz. prescription vials, white glass, at a temperature of 65°. We noted the following consequences:

In No. 1 the casein slowly separated from the whey, *and settled upon the bottom of the vial*, the precipitation being not quite complete.

In No. 3 we observed small granules of precipitated casein, smaller and compacter than those in No. 1, but the fluid retained its milky appearance.

In No. 2 the casein precipitated completely, *i. e.*, as far as we could observe from the physical appearance of the vial contents, but the *coagulum was decidedly more flocky*, *and*, *what is very remarkable*, *it floated upon the clear whey.*

To be sure that no unforseen interference caused this difference, we repeated the experiment with Nos. 1 and 2; taking, this time, three ounces of fresh milk, and one ounce of the same vinegar, adding to No. 2 one drachm of sweet oil of almonds, with positively the same consequences. The only difference, perhaps, being a more distinct separation of the casein in No. 1, owing to the increased amount of vinegar added. In No. 1 the whey was above the precipitate, and in No. 2 it was below the coagulum.

In both experiments there was a separation of two distinct precipitates, one floating upon the whey, and the other settling on the bottom of the vial, this phenomenon being observed only in the mixture that contained the oil, and so fixed is this separation, that after repeated shaking the same division would take place.

The result of these experiments, though expected, demonstrated to us the fact that the looser coagulum of woman's milk is formed by the greater amount of fat in it (compared with the per centage of casein), and that this physical condition can be artificially produced by the addition of one scruple of oil of sweet almonds or cod-liver oil to every two ounces of cows' milk. It is proven that the presence of fat is necessary for the digestion of protein bodies; it is known that a larger amount will facilitate this process (of course not in a quantity above normal requirements.) We took a half drachm of pepsin, added to it one and one-half ounces of water and acidulated the mixture with twenty drops of muriatic acid; after some time we added of the clear fluid, avoiding the white precipitate on the bottom of the bottle, one-half ounce to each vial of our first series of experiments, and find, twenty-four hours after the admixture, in a temperature of sixty-eight degrees, half of the precipitate in No. 2 redissolved, whereas it is hardly attacked in No. 3 and but little in No. 1, proving that even out of the body the above statement is true.

Elsaesser has demonstrated the fact that the easier digestion of the fibrine in woman's milk is caused by the gelatinous, light texture of its coagulum; and

We think we have ascertained *that it is the larger amount of fat mixed with the fibrine in woman's milk, that causes the spongy consistence of the coagulate, and*

WE JUSTLY CLAIM, THAT THE ADMIXTURE OF FAT WITH COWS' MILK WILL MAKE THIS THE BEST ARTIFICIAL FOOD FOR INFANTS.

Should our deductions not meet with the entire approval of the profession at large, of which we have no sanguine expectation, we nevertheless earnestly hope that this article will help to shake the positively untrue opinion, that cows' milk will be a good substitute for mothers' milk when diluted with water and with the addition of sugar.

We think experience has amply proven how watery this water-doctrine is, and we exclaim, with the great Lehmann,

"Spring must yet come."

Will the friends of progress be kind enough and give this formula of ours a fair trial, by instructing the nurses and mothers of infants, not only as to the manner of its use, but explain to them also its mode of action, capacitating them, as much as possible, to nurse their children understandingly; for then, and only then, will

"The hand that rocks the cradle, be the hand that rocks the world."

